# Community assembly in Lake Tanganyika cichlid fish: quantifying the contributions of both niche‐based and neutral processes

**DOI:** 10.1002/ece3.2689

**Published:** 2017-01-22

**Authors:** Thijs Janzen, Adriana Alzate, Moritz Muschick, Martine E. Maan, Fons van der Plas, Rampal S. Etienne

**Affiliations:** ^1^ Department of Evolutionary TheoryMax Planck Institute for Evolutionary BiologyPlönGermany; ^2^ Groningen Institute for Evolutionary Life SciencesUniversity of GroningenGroningenThe Netherlands; ^3^ Terrestrial Ecology UnitUniversity of GhentGhentBelgium; ^4^ Fundacion EcomaresCaliColombia; ^5^ Zoological InstituteUniversity of BaselBaselSwitzerland; ^6^ Department of Fish Ecology & EvolutionEAWAG Centre for EcologyKastanienbaumSwitzerland; ^7^ Institute of Plant SciencesUniversity of BernBernSwitzerland; ^8^ Biodiversity and Climate Research CentreSenckenberg Gesellschaft für NaturforschungFrankfurtGermany

**Keywords:** cichlids, Lake Tanganyika, STEPwise Community Assembly Model, trait‐based community assembly

## Abstract

The cichlid family features some of the most spectacular examples of adaptive radiation. Evolutionary studies have highlighted the importance of both trophic adaptation and sexual selection in cichlid speciation. However, it is poorly understood what processes drive the composition and diversity of local cichlid species assemblages on relatively short, ecological timescales. Here, we investigate the relative importance of niche‐based and neutral processes in determining the composition and diversity of cichlid communities inhabiting various environmental conditions in the littoral zone of Lake Tanganyika, Zambia. We collected data on cichlid abundance, morphometrics, and local environments. We analyzed relationships between mean trait values, community composition, and environmental variation, and used a recently developed modeling technique (STEPCAM) to estimate the contributions of niche‐based and neutral processes to community assembly. Contrary to our expectations, our results show that stochastic processes, and not niche‐based processes, were responsible for the majority of cichlid community assembly. We also found that the relative importance of niche‐based and neutral processes was constant across environments. However, we found significant relationships between environmental variation, community trait means, and community composition. These relationships were caused by niche‐based processes, as they disappeared in simulated, purely neutrally assembled communities. Importantly, these results can potentially reconcile seemingly contrasting findings in the literature about the importance of either niche‐based or neutral‐based processes in community assembly, as we show that significant trait relationships can already be found in nearly (but not completely) neutrally assembled communities; that is, even a small deviation from neutrality can have major effects on community patterns.

## 1 Introduction

The stunning diversity of cichlid fishes in the African Rift lakes has fascinated scientists for decades (Brooks, 1950; Coulter, 1991; Fryer & Iles, 1972; Kocher, 2004; Wagner, Harmon, & Seehausen, 2012). In contrast to the large body of research focusing on the evolutionary explanations of cichlid diversity (Brawand et al., 2014; Genner & Turner, 2011; Joyce et al., 2011; Magalhaes, Mwaiko, Schneider, & Seehausen, 2009; Muschick, Indermaur, & Salzburger, 2012; Sturmbauer, Salzburger, Duftner, Schelly, & Koblmüller, 2010; Wagner et al., 2013), there are fewer studies aiming at understanding the ecological mechanisms responsible for local coexistence and community diversity. Empirical studies on local scale diversity have focused either on temporal trends (Hori, Gashagaza, Nshombo, & Kawanabe, 1993; Takeuchi, Ochi, Kohda, Sinyinza, & Hori, 2010), the impact of human disturbance (Alin, Cohen, & Bills, 1999), opportunities for relieving fishing efforts (Duponchelle, Ribbink, Msukwa, Mafuka, & Mandere, 2003; Weyl, Nyasulu, & Rusuwa, 2005), the impact of protected areas on cichlid communities (Sweke, Assam, Matsuishi, & Chande, 2013), or have been restricted to descriptions only (Hori, Yamaoka, & Takamura, 1983; Kuwamura, 1987; Van Steenberge et al., 2011). These studies have identified several ecological and nonecological factors influencing local species diversity. Here, we quantify the contributions of both ecological and nonecological processes to variation in community composition.

Community assembly occurs on a continuum between a niche‐based perspective and a neutral perspective (Gravel, Canham, Beaudet, & Messier, 2006; Kalyuzhny et al., 2014; Wennekes, Rosindell, & Etienne, 2012). The niche‐based hypothesis postulates that species are adapted to their local environment and occupy a specific niche: a set of conditions in which the species thrives and outcompetes other species (Chesson, 2000; Hutchinson, 1959; Tilman, 1982). The traits of a species reflect its adaptation to its niche, and studying patterns in community trait distributions can inform us about underlying processes driving species coexistence and community composition. In benign environments that do not pose strong requirements on traits, the niche‐based hypothesis predicts that species richness is high and that the presence or absence of species with particular traits is the result of species interactions, rather than interactions with the abiotic environment. Due to the exclusion of species with overlapping niches, with shared specialist predators, or with shared parasites, niche‐based assembly is expected to generate high trait diversity among co‐occurring species in benign environments (Macarthur & Levins, 1967; Mayfield & Levine, 2010). In harsh environments, the niche‐based hypothesis predicts low species richness, and predicts that species with traits that make them intolerant to stress, herbivory, and/or predation pressures might be excluded from a local community, generating lower trait diversity among co‐occurring species (Cornwell, Schwilk, & Ackerly, 2006; Weiher & Keddy, 1995).

In contrast, the neutral hypothesis, which considers all individuals from all species as equivalent, explains community composition by stochastic processes, where the local abundance of a species is the outcome of stochastic birth, death, and migration over time (Hubbell, 2001; Rosindell, Hubbell, & Etienne, 2011; Rosindell, Hubbell, He, Harmon, & Etienne, 2012). Local community composition is assumed to be a dynamic equilibrium between random immigration from the species pool and local ecological drift. The neutral hypothesis acknowledges that there might be benign and harsh environments, but that these environments affect all individuals equally. As a consequence, benign environments have more individuals than stressful environments, but both benign and stressful areas contain individuals that form a (dispersal‐limited) random subset of the species pool. The null expectation is then that areas with high abundances also have high species richness, as a result of random sampling.

Previous attempts to assess community assembly have focused on analyzing a single process at a time: limiting similarity (Kursar et al., 2009), habitat filtering (Cornwell et al., 2006; Kraft, Cornwell, Webb, & Ackerly, 2007), or stochastic community assembly (Etienne & Alonso, 2005). Recently, we have developed a theoretical framework that can jointly estimate the contributions of limiting similarity, habitat filtering, and species‐neutral stochasticity: STEPCAM (STEPwise Community Assembly Model) (Van der Plas et al., 2015). Here, we apply this approach to communities of African lake cichlids, textbook examples of adaptive radiation, and niche segregation. In addition to divergent trophic adaptation (Fryer & Iles, 1972; Kocher, 2004; Konings, 2005), lacustrine cichlid species often segregate along depth gradients (Ribbink, Marsh, Marsh, Ribbink, & Sharp, 1983; Seehausen & Bouton, 1997), entailing various adaptations including trophic morphology and sensory abilities. In South American rivers, cichlids are known to experience high levels of limiting similarity, also suggesting an important role for niche‐based processes (Montaña & Winemiller, 2010; Montaña, Winemiller, Sutton, & Inemiller, 2014). Furthermore, habitat complexity has been shown to be an important driver of niche processes, as complex habitats are often associated with a reduction in territoriality (Danley, 2011), a larger number of niches (Willis, Winemiller, & Lopez‐Fernandez, 2005) and higher diversity (Ding, Curole, Husemann, & Danley, 2015). Thus, cichlids are a promising case study to unravel the quantitative contributions of niche‐based and neutral processes, and to infer to what extent community assembly is driven by these processes.

## 2 Methods

### 2.1 Abundance data

**Figure 1:**
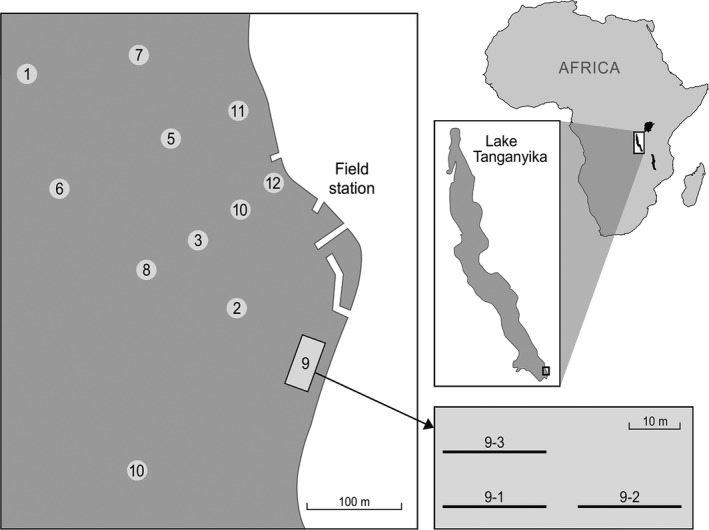
Sampling positions in front of the Kalambo Lodge, located in the south of Lake Tanganyika. Relative position of the transects in every sampling cluster is indicated in the left panel. Numbers in the figure refer to cluster numbers in Tables S1–S3

Abundance and community composition data of cichlids were collected in Lake Tanganyika, near Kalambo Lodge (8°37′22.29″S, 31°12′1.89″E), Zambia, Africa (Figure 1), using scuba diving. In total, 36 transects were sampled, grouped in sampling clusters of 3. Transects were placed parallel to the shore (Figure 1). Individuals were visually recorded along 20 m × 4 m transects by two divers in two steps: First, all individuals within 2 m on one side of the transect were sampled. After 10 min, all individuals within 2 m on the other side of the transect were sampled. Sampling was performed nonintrusively through visual identification. We defined the local community as all individuals observed along one transect.

### 2.2 Environmental data

At each transect, we measured three different ecological variables. First, we recorded the depth at the beginning and the end of each transect. Second, we took 40 photographs of 50 × 50 cm quadrats per transect, to estimate the percentage of sand cover (20 photographs on one side of the transect line and 20 photographs on the other side). Percentage of sand cover per quadrat was calculated using an image analysis script in MATLAB (Supporting Information). The variation of sand cover was lower within transects than between sand transects (standard deviation within transects: 17%, between transects: 26%); thus, we used the average proportion of sand across all photographs taken along the transect to quantify sand cover of each transect. Third, we recorded the topographical complexity of the substrate using a variation of the “chain method” (Risk, 1972). Along the transect, a link‐brass chain was laid over the substrate, such that the chain would closely follow the contour of the substrate (Shumway et al., 2007). Topographical complexity was calculated as the ratio between the distance following the contour (measured with the chain) and the horizontal linear distance. High values indicate high relief/complexity, caused by an alternation between large rocks and small rocks or sand, while low values indicate low relief/complexity.

### 2.3 Species traits

Data on mean trait values of each cichlid species as reported by Muschick et al. (2012, 2014) were collated for 10 traits: standard length, total length, weight, stable isotope ratios of carbon and nitrogen, lower pharyngeal jaw height, lower pharyngeal jaw width, gut length, lower pharyngeal jaw shape, and body shape. To obtain a set of traits that do not strongly correlate with each other, to avoid the overemphasis of the importance of some traits over others and to avoid pseudoreplication, we only used a subset of these traits in our STEPCAM analysis. The final trait set used for STEPCAM consisted of six traits: standard length, stable isotope ratios of carbon and nitrogen, gut length, the first PCA component of LPJ shape, and the first PCA component of body shape. Further details on our trait selection procedure can be found in the supplementary material.

### 2.4 Trait‐based community assembly

To infer the contribution of limiting similarity, habitat filtering and stochasticity to community assembly, we used the STEPCAM approach (Van der Plas et al., 2015). The STEPCAM model is a STEPwise Community Assembly Model that applies three types of processes to select species from the species pool into the local community. Starting with all observed species in the dataset, species are removed in a stepwise fashion until reaching the number of species actually observed in the local community. Removal of species occurs either (1) because their traits are too dissimilar from the observed mean trait distribution in the community, which is assumed to be the habitat optimum (“filtering”), (2) because their traits are too similar to the other remaining species (“limiting similarity”), or (3) due to a stochastic event, which results in a random removal step, where the probability of removal is negatively proportional to the number of local communities in the dataset where the species is observed, which is used as a proxy for the species pool. The STEPCAM model was fitted using approximate Bayesian computation, in which, using the model, data are simulated and compared with the observed data. Comparison between simulated and observed data occurred through comparing four summary statistics: functional richness, functional evenness, functional divergence, and community trait means (Van der Plas et al., 2015; Villéger, Mason, & Mouillot, 2008). We applied a sequential Monte Carlo algorithm (ABC‐SMC) using the function STEPCAM_ABC from the package STEPCAM (Janzen & van der Plas, 2016). We used 1,000 particles and a final acceptance rate of 1 in 20,000. The reported estimates for stochasticity, filtering, and limiting similarity are mean estimates over three replicate STEPCAM runs, with the random number generator seeded with different seeds for each replicate. Reported are the number of steps relative to the total number of steps, in percentages. This allows for the comparison of contributions of the three processes across different transects with different species richness.

### 2.5 The effect of the environment on community trait means

To test whether variation in trait values was related to variation in environmental variables, we calculated, per transect, community‐level trait means (CTM). We used linear mixed models to test how CTM values correlated with environmental variation. We constructed full models, where CTM values were treated as dependent variable, the three habitat characteristics as fixed effects, and the transect cluster as a random effect. Nonsignificant predictor variables were then removed in a stepwise fashion. Both dependent variables and fixed effects were scaled before applying the linear mixed models, in order to obtain standardized regression coefficients. Conditional *R* ^2^ values were calculated following Nakagawa and Schielzeth (2013).

To assess the effect of environmental variation on mean trait values in the absence of niche‐based processes, we simulated artificial communities using STEPCAM, in the absence of any niche‐based processes. Hence, we simulated an artificial community for each transect with the contributions of limiting similarity and habitat filtering set to 0% and the contribution of stochasticity set to 100%. We then calculated mean trait values for each of these artificial communities and investigated their relationship with environmental variables using linear mixed models with the same predictor variables as the finally selected models explaining mean trait values of the empirical data. We repeated this procedure 100 times and calculated for each trait the average effect size of each predictor across the hundred models and the average model *R* ^2^. If niche‐based processes are important drivers of the observed environmental trait relationships, we expect that in the absence of niche‐based processes, the average effect sizes of the predictor variables reduce and that the average *R* ^2^ values become lower.

To assess to what extent simulated communities reflect the same mean trait patterns as the observed communities, we used the estimated contributions of stochasticity, habitat filtering, and limiting similarity and simulated artificial communities using STEPCAM. The fit of simulated communities was compared to the final fit obtained for the empirical data, and only communities having a similar, or better, fit than accepted parameter values in the last iteration of the STEPCAM optimization were accepted. We simulated 100 such well‐fitting artificial communities per transect, on which we applied the linear mixed models, obtained from the empirical data, to assess the relationship between CTMs of these artificial communities with the environment. We report the average predictor variable values and the average *R* ^2^ across these 100 communities.

### 2.6 Community dissimilarity

To assess the simultaneous effect of all three environmental characteristics on community composition, we quantified community dissimilarity (Bray–Curtis dissimilarity) between all communities. We constructed an environmental distance score by calculating the distance between all transects for each environmental characteristic (depth, sand, and complexity). To ensure that all environmental factors had a similar weight on environmental heterogeneity, we normalized the distance scores by the maximum distance, such that all individual distance scores were between −1 and 1. We obtained the total normalized environmental distance by taking the square root of the sum of squared distance scores. We then correlated both distances with each other using linear regression and assessed significance using a Mantel test. To assess the relationship between environmental dissimilarity and community dissimilarity in the absence of niche‐based processes, we simulated 100 artificial communities using STEPCAM for each transect, with the contribution of limiting similarity and filtering processes set to 0% and the contribution of stochastic processes set to 100%. This way, at each transect, a species would occur in a minimum of 0 artificial communities and a maximum of 100 artificial communities. The average frequency of each species in each transect (between 0 and 1) was used as a measure of its relative abundance in the artificial communities. We calculated community dissimilarity between the artificial communities and correlated that with environmental dissimilarity. If niche‐based processes are important in driving relationships between environmental dissimilarity and community dissimilarity, then we would expect that in the absence of niche‐based processes, the relationship between environmental dissimilarity and community dissimilarity would become much weaker.

## 3 Results

### 3.1 Species compositions

We recorded on average 137 individuals and 22 species per transect, an accumulated total of 4,926 individuals and 49 species (Table S3). *Telmatochromis temporalis* was the most common species, contributing 12% of 4,926 recorded individuals. The seven most common species combined accounted for 50% of all observed individuals (*T. temporalis*,* Variabilichromis moorii*,* Tropheus moorii*,* Neolamprologus pulcher*,* Interochromis loocki*,* Telmatochromis vittatus*, and *Xenotilapia boulengeri*), while the 24 most common species accounted for 90% of all individuals.

**Figure 2:**
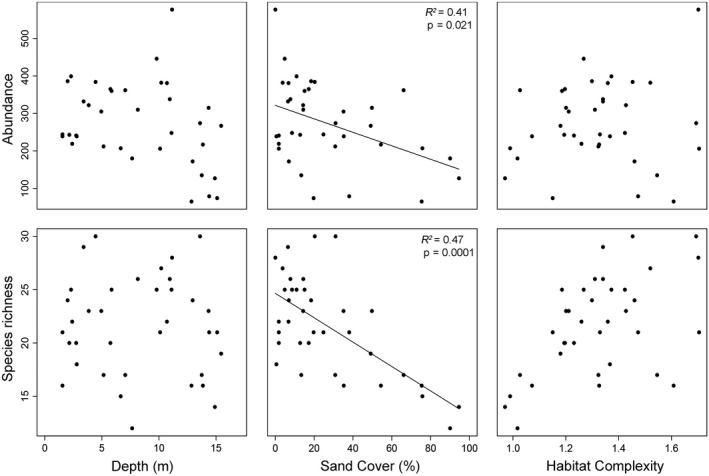
Abundance and species richness against three environmental characteristics. Points depict the different transects. Significant correlations are plotted as a line

Transects with a higher sand cover had a lower number of individuals (*R* ^2^ = .41, *p* = .021) and a lower species richness (*R* ^2^ = .47, *p* = .001) (Figure 2). Neither depth nor habitat complexity had a significant effect on abundance or species richness (Figure 2). Depth, sand cover, and habitat complexity did not significantly correlate with each other (nonlinear mixed model, transect cluster as random factor, all *p*‐values >.05).

**Figure 3:**
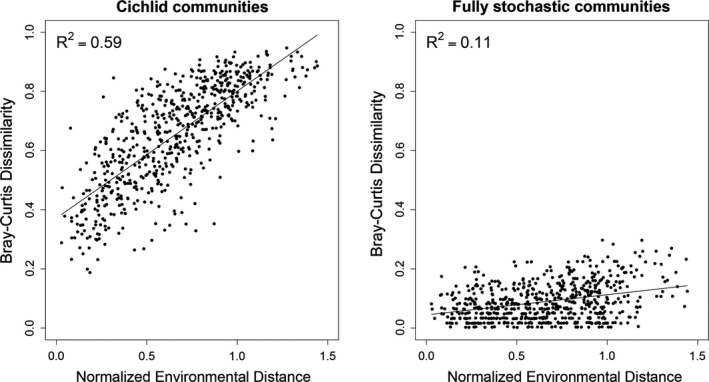
Changes in Bray–Curtis dissimilarity between transects versus the normalized environmental distance between sampled transects. Normalized environmental distance is the Euclidian distance between two transects, where the environmental distances are normalized by the maximum value recorded across all transects. Left panel shows community dissimilarity between observed communities (*R* ^2^ = .59, Mantel‐*r* = 0.766, *p* < .001); right panel shows community dissimilarity between communities simulated without niche‐based effects (*R* ^2^ = .19, Mantel‐*r* = .335, *p* < .001)

Bray–Curtis dissimilarity between transects was found to correlate significantly with the normalized environmental distance score (Figure 3, left panel, *R* ^2^ = .59, Mantel‐*r* statistic = .766, *p* = .00001, 100,000 permutations), indicating that similar habitats harbored similar species communities. Bray–Curtis dissimilarity of communities simulated using STEPCAM in the absence of niche‐based processes also correlated significantly with normalized environmental distance, but had a much lower *R* ^2^ value (Figure 3, right panel, *R* ^2^ = .11, Mantel test, Mantel‐*r* statistic = .335, *p* = .00001, 100,000 permutations), and a significantly lower slope (slope for empirical communities: 0.43, for simulated communities: 0.07, ANCOVA, *p* < 2e‐16), indicating that environmental variation has a highly significant effect on community composition.

### 3.2 Contributions of different community assembly processes

**Figure 4:**
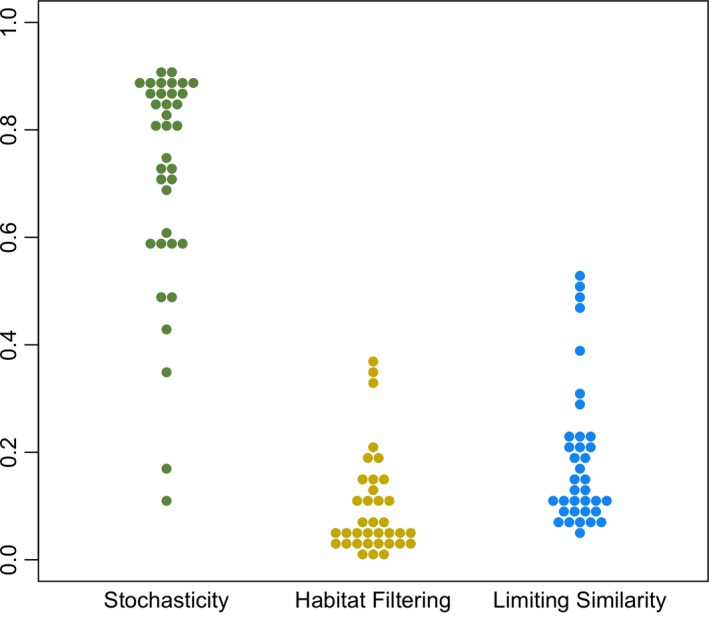
Contributions of stochasticity, habitat filtering, and limiting similarity steps across all 36 transects. Each dot is the mean estimate across three independent STEPwise Community Assembly Model (STEPCAM) inferences per transect

**Figure 5:**
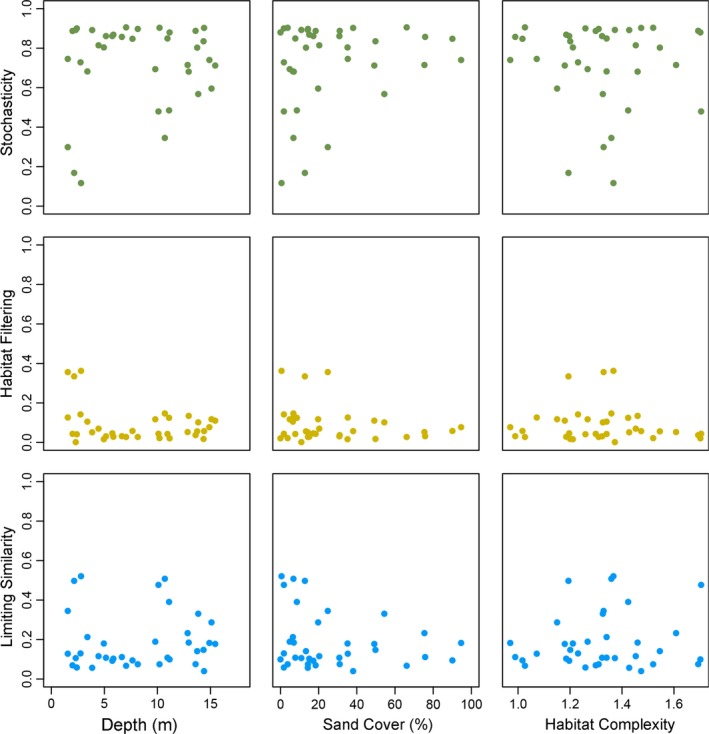
Contributions of stochasticity, habitat filtering, and limiting similarity steps as estimated using STEPwise Community Assembly Model (STEPCAM), plotted against the three measured habitat characteristics: depth, sand cover, and complexity. None of these relationships were significant

Fitting STEPCAM to the trait distributions of the 36 different transects yielded an average contribution of stochastic assembly steps of 72%, an average contribution of habitat filtering steps of 9%, and an average contribution of limiting similarity steps of 19% (Figure 4). We found no significant correlations between the contributions of any of the three processes and any of the three habitat characteristics (Figure 5), indicating that the relative importance of the three processes of community assembly did not differ between habitat types.

### 3.3 Relationships between traits and habitat characteristics

We found that the three habitat characteristics explained a significant proportion of variation in community trait means (CTMs) (Table 1). We observed high *R* ^2^ values (*R* ^2^ > .70) for δ^15^N content, δ^13^C content, and the first axis of the PCA of the lower pharyngeal jaw shape (Table 1). Across all traits, the majority of linear mixed models included depth as a significant predictor variable, which also often had the largest regression coefficient.

**Table 1:** Significant predictor variables of linear mixed‐effects models, where mean species traits per transect were used as response variables, habitat characteristics as predictor variables, and sampling cluster as a random effect

Response variable	Empirical transects				Best fitting simulated transects, mean values of 100 replicates				Fully stochastic simulated transects, mean values of 100 replicates			
	Depth	Sand	Complexity	*R* ^2^	Depth	Sand	Complexity	*R* ^2^	Depth	Sand	Complexity	*R* ^2^
Traits included in STEPCAM												
Standard length	−0.50	0.42	0.57	.54	−0.41	0.31	0.53	.35	0.002	−0.195	0.069	.19
δ^15^N	0.77			.76	0.75			.70	−0.028			.10
δ^13^C	−0.71	−0.29		.78	−0.65	−0.38		.74	0.011	0.043		.14
Gut length	−0.73		0.44	.58	−0.74		0.44	.60	−0.042		0.176	.12
LPJ PCA 1	−0.54	−0.39	−0.36	.78	−0.60	−0.30	−0.26	.70	0.014	0.049	−0.077	.19
Body PCA 1	0.44	0.46		.66	0.37	0.53		.58	−0.044	0.163		.13
Other traits												
Total length			0.46	.48			0.31	.25			0.192	.14
Weight	−0.54		0.36	.58	−0.44		0.40	.31	−0.077		0.172	.12
LPJ height	−0.41			.32	−0.36			.44	−0.024			.09
LPJ width	−0.39			.29	−0.10			.08	−0.021			.09
LPJ PCA 2	−0.74		0.31	.54	−0.74		0.36	.59	−0.008		0.122	.13
LPJ PCA 3		−0.34	0.47	.51		−0.29	0.07	.26		−0.126	0.070	.15
Body PCA 2	0.78	−0.27		.67	0.55	−0.27		.51	0.059	−0.269		.18
Body PCA 3			−0.78	.60			−0.34	.14			−0.168	.12

Response and predictor variables were scaled, to allow for comparison between components. Unscaled components can be found in the supplementary material. Only those components that were significant after stepwise removal of all nonsignificant components are reported. Conditional *R* ^2^ of the final model is reported in the last column. The first six rows provide information on traits used in the STEPCAM analysis; the other rows provide information on other traits available in the dataset. Shown are significant components for the transect data, mean components over 100 replicate artificial communities generated using best STEPCAM estimates, and mean components over 100 replicate artificial communities generated using solely stochastic community assembly. LPJ, lower pharyngeal jaw, PCA, principal component axis.

When applying linear mixed models on community trait means of artificial communities generated using mean STEPCAM estimates, we found very similar estimates as for the empirical data (Table 1). We observed high *R* ^2^ values for δ^15^N content (*R* ^2^ = .70), δ^13^C content (*R* ^2^ = .74), and lower pharyngeal jaw shape (*R* ^2^ = .70). Estimates were especially similar for traits included in the STEPCAM analysis (standard length, δ^15^N content, δ^13^C content, gut length, the first PCA component of LPJ shape, and the first PCA component of body shape), whereas traits not included in the STEPCAM analysis tended to have a lower *R* ^2^. The recovery of similar regression estimates indicates that the communities reconstructed by STEPCAM resemble those actually observed in terms of trait composition.

Correlating the three habitat characteristics with community trait means of communities generated using solely stochastic species removal, we found that across all traits, *R* ^2^ were low and that all regression coefficients were close to zero. This suggests that the inclusion of habitat filtering and limiting similarity is imperative for these relationships and demonstrates that in the absence of niche‐based processes, such relationships vanish.

## 4 Discussion

We have investigated whether community assembly in cichlid communities in the littoral zone of Lake Tanganyika, Zambia, is mostly driven by niche‐based processes or by neutral‐based processes. We found that across all environmental characteristics, neutral‐based processes were responsible for the majority of community assembly. The strong relationships between average trait values and environmental characteristics suggest that even though niche‐based processes only contributed a minority of all community assembly steps in the STEPCAM model, their influence on average trait values and community composition was high.

The average contribution of niche‐based processes to community assembly was relatively low, suggesting their role to be minor. However, when we repeated our analysis of trait means for communities simulated in the absence of niche‐based processes, we found that correlations between community trait means and local environmental conditions disappeared. Thus, although niche‐based processes altogether were only responsible for 28% of all community assembly steps, they significantly shaped communities, both in terms of species composition and in terms of mean trait values. This is an important finding, as it might resolve seemingly contrasting findings in the literature, where some studies emphasized the importance of stochastic processes based on species abundance distributions or species area relationships (Condit, Hubbell, & LaFrankie, 1996; Hubbell, 2001; Rosindell & Cornell, 2009), while other studies pointed at the importance of niche processes based on trait–environment relationships (Cavender‐Bares, Kitajima, & Bazzaz, 2004; Cornwell & Ackerly, 2009; Kraft, Valencia, & Ackerly, 2008). Our study shows that even when stochastic processes are responsible for the majority of community assembly steps, a small contribution of niche‐based processes can already cause significant trait–environment relationships.

The low proportion of niche‐based processes identified by STEPCAM may be due to our choice of traits. In an ideal scenario, one would include information on all possible traits. However, some traits are hard to measure, and empirical support for the functional importance of traits is an ongoing process (e.g., acoustic diversity (Danley, Husemann, & Chetta, 2012; Spinks, Muschick, Salzburger, & Gante, 2016). Hence, it seems likely that we have missed some relevant traits and that some niche axes that are potentially important to explain diversity are not included in our analysis. In order to minimize this effect, we have focused here on traits associated with shifts in diet (total length, gut length, pharyngeal jaw shape, and body shape), which are known to differ most between habitats, as they are related to trade‐offs regarding resource uptake (McGee et al., 2015; Muschick et al., 2012). Furthermore, we have included stable isotope ratios of carbon and nitrogen, which are known to reflect trophic level and food type (Muschick et al., 2012). Our choice of traits therefore focuses on adaptations related to food uptake and diet, which we expected to be important drivers of diversity. An interesting future extension of our work would be the inclusion of traits linked to coloration and visual adaptation, which are associated with several fitness‐determining processes. For example, male coloration has been shown to be spatially overdispersed in cichlids (Seehausen & Schluter, 2004), presumably due to limiting similarity as a result of color‐dependent aggression (Dijkstra, Seehausen, Pierotti, & Groothuis, 2007). Cichlid coloration is also expected to be correlated with the local habitat, with vertical bar patterns being advantageous in complex habitats (Seehausen, Mayhew, & Alphen, 1999), and mimicry or crypsis helping to obtain resources (Boileau et al., 2015; Schelly et al., 2007) or evade predation (Seehausen et al., 2008). Lastly, variation in coloration and visual perception has been shown to be associated with depth segregation and to be important factors in mate choice and species divergence (Miyagi, Terai, Aibara, & Sugawara, 2012; Seehausen, van Alphen, & Witte, 1997). The inclusion of traits associated with coloration and visual adaptation could therefore capture niche dimensions that are not included in the current analysis. This may lead to an increase in the importance of limiting similarity, although depth segregation could manifest itself through heightened habitat filtering as well. Furthermore, a comparison between estimates obtained using only diet‐associated traits, using only traits associated with coloration and visual adaptation, or the combination of both these types of traits might partition the causes of diversity in causes directly related to diet, and causes more related to sexual selection, an ongoing debate in the literature (Doorn, Noest, & Hogeweg, 1998; Kocher, 2004; Maan & Seehausen, 2011; Oneal & Knowles, 2013; Seehausen et al., 2014; Sobel, Chen, Watt, & Schemske, 2010).

The large contribution of neutral processes in community assembly observed here is in line with previous findings focusing on factors influencing cichlid diversity from a macro‐evolutionary (rather than an ecological) perspective. The neutral theory predicts that the number of species within a community is directly related to the total number of individuals in a community, because communities with a large number of individuals are more likely to accumulate speciation events (Rosindell & Cornell, 2009). Using advanced regression techniques, Wagner et al. (2012), Wagner, Harmon, and Seehausen (2014) showed that the probability of success, and the resulting diversity, of a lacustrine cichlid radiation depends on multiple extrinsic environmental factors, including the depth, the size, and the total received solar input of a lake. These findings are consistent with our observations that both depth and stochasticity play major roles in community assembly. Depth, size, and total received solar input of a lake are among the main drivers behind the maximum number of individuals that a lake can sustain. The maximum number of individuals in turn then determines the number of species following neutral theory. Wagner et al. (2012) also found significant effects of intrinsic lineage‐specific traits on radiation potential, including sexual dichromatism and sexual dimorphism. This iterates the potential of including traits associated with coloration and sexual selection in future community assembly studies as well.

Summarizing, we find conflicting results, with on the one hand STEPCAM estimates pointing toward an important role for stochastic processes and on the other hand strong relationships between environmental variation, trait means, and community composition. Our conflicting results resonate the ongoing debate attempting to discern the underlying processes of community assembly (Wennekes et al., 2012), with explanations emphasizing either stochastic processes (Hubbell, 2001; Rosindell et al., 2011, 2012) or niche‐based processes (Cornwell & Ackerly, 2014; HilleRisLambers, Adler, Harpole, Levine, & Mayfield, 2012; Kraft et al., 2007, 2008; Van der Plas, Anderson, & Olff, 2012). Here, we find that niche processes, although only responsible for a minority of community assembly steps, are responsible for the majority of trait‐based patterns. We conclude that community assembly is driven both by niche and neutral processes simultaneously—which likely holds for many other taxa as well (Dumbrell, Nelson, Helgason, Dytham, & Fitter, 2010; Lee, Buckley, Etienne, & Lear, 2013; Rominger, Miller, & Collins, 2009). Our results suggest that niche‐based processes exert their influence well beyond their quantitative contribution to the whole community assembly process, generating strong relationships between environmental variation, trait variation, and community composition. Lastly, our findings resonate previous findings in savannah trees, suggesting that observed relative contributions of niche versus neutral‐based processes are not specific to cichlids alone, but might prove to be a more general trend across communities. Future work extending the joint estimation of niche and neutral processes toward other communities might show to what extent these patterns are general, and to what extent other communities support our findings.

## Conflict of Interest

None declared.

## Author Contributions

TJ, AA, MEM and RSE designed the study. TJ and RSE acquired funding supporting the fieldwork. Fieldwork was performed by TJ and AA. Fieldwork data was combined with data obtained previously by MM, and analyzed using STEPCAM by TJ and FvdP. A first draft of the manuscript was made by TJ, upon which AA, MM, MEM, FvdP and RSE improved iteratively.

## Data Availability

Sand cover, raw count data, and STEPCAM output files for all transects are available on data dryad: http://dx.doi.org/10.5061/dryad.d1s39.
