# Supplementary material for: Community assembly in Lake Tanganyika cichlid fish: quantifying the contributions of both niche‐based and neutral processes
Source: Ecol Evol. 2017 Jan 22;7(4):1057–67. doi: 10.1002/ece3.2689 (PMC5306054; doi:10.1002/ece3.2689)
Supplement: Supplementary file 4 [file ECE3-7-1057-s004.docx]

SUPPLEMENTARY MATERIAL

*Selection of traits*

In order to apply STEPCAM, we collated mean trait values of each cichlid species as reported by Muschick and colleagues (2012, 2014) for 11 traits: standard length, total length, weight, stable isotope ratios of carbon and nitrogen, lower pharyngeal jaw height, lower pharyngeal jaw width, gut length, lower pharyngeal jaw shape and body shape. First we checked the Pearson Rank correlation between traits. Total length and weight correlated strongly with standard length (standard length vs total length: *R^2^* = 0.98, standard length vs weight: *R^2^* = 0.75), and we decided to only use standard length. Furthermore, lower pharyngeal jaw (LPJ) width and lower pharyngeal jaw height were correlated with each other (*R^2^* = 0.67) and with standard length (LPJ height vs standard length: *R^2^* = 0.44, LPJ width vs standard length: *R^2^* = 0.37). To avoid overemphasis of pharyngeal jaw traits, and because of the high correlation coefficients we decided to omit both these traits. Lower pharyngeal jaw shape and body shape were assessed using landmark-based geometric morphometric methods. *xy* coordinates of the landmarks were combined into PCA components for both body shape and lower pharyngeal jaw shape. From these PCA components we included the first PCA component for both body and lower pharyngeal jaw shape, which contained 37% and 49% of the variation for body and pharyngeal jaw shape respectively.

The final trait set thus consisted of six traits: standard length, stable isotope ratios of carbon and nitrogen, gut length, the first PCA component of LPJ shape and the first PCA component of body shape. Missing trait values (δ15N and δ13C for *Neolamprologus caudopunctatus, N. cylindricus & Ophthalmotilapia nasuta*, and gut length for *N. cylindricus, Simochromis babaulti* and *Telmatochromis vittatus*) were imputed using the MICE package for R (Buuren & Groothuis-Oudshoorn 2011). The MICE imputation process uses a Gibbs sampler technique to impute the missing data, assuming a multivariate distribution for the missing data.

References:

Buuren, S. & Groothuis-Oudshoorn, K. (2011). MICE: Multivariate imputation by chained equations in R. *Journal of Statistical Software*, **45**.

Muschick, M., Indermaur, A. & Salzburger, W. (2012). Convergent evolution within an adaptive radiation of cichlid fishes. *Current Biology*, **22**, 2362–8.

Muschick, M., Nosil, P., Roesti, M., Dittmann, M.T., Harmon, L. & Salzburger, W. (2014). Testing the stages model in the adaptive radiation of cichlid fishes in East African Lake Tanganyika. *Proceedings of the Royal Society B*, **281**, 1–13.
